# Results of upper cervical palpation test in migraine patients are not explained by pain sensitization

**DOI:** 10.1038/s41598-025-03344-6

**Published:** 2025-06-25

**Authors:** Juliana Pradela, Melanie Maria Otto, Emily Kaske, Luise John, Nicole Derner, Arne Vielitz, Kerstin Luedtke

**Affiliations:** 1 Department of Health Science, University of São Paulo, São Paulo, Brazil; 2 Department of Physiotherapy, Pain and Exercise Research Luebeck (P.E.R.L), Institute of Health Sciences, University of Luebeck, Lübeck, Germany

**Keywords:** Migraine, Neck pain, Neck-related disability, Pain sensitization, Upper cervical spine sensitivity, Palpation, Outcomes research, Neurological disorders

## Abstract

The exact cause for no pain, local pain and referred pain groups according to the upper cervical palpation test (UPT) and whether neck pain in migraine patients is caused due to pain sensitization or influenced by perceived neck-related disability, is not fully understood. The aim was to determine whether upper cervical spine sensitivity tested by the UPT is associated with neck-related disability or increased pain sensitization in patients with migraine. Forty-two patients with episodic migraine were examined regarding mechanical and pressure pain thresholds, central sensitization (CSI), allodynia (ASC-12) and neck-related disability (NDI), and sub-grouped according to the UPT. An ANOVA analysis was performed for group differences. Exploratory regression and correlation analyses were performed with NDI and CSI as dependent variables to understand which factors are related and contribute to either subgroup allocation. No significant differences were found in UPT subgroups regarding CSI and NDI. The UPT subgroups could not be determined by any evaluated variable. The NDI was explained in 43.6% by the CSI and neck pain intensity. CSI results were explained to 49.4% by a model including ASC-12 and NDI. In conclusion, UPT subgroups were neither explained by differences in CSI, mechanical or pressure threshold testing or NDI.

## Introduction

Migraine is one of the diseases with the greatest global burden. In women and young adult men, headache disorders, especially migraine, are ranked among the top 10 diseases leading to disability in daily life^1^. Migraine is assumed to have a neurobiological cause originating in the central nervous system^2^. The biological cascade associated with a migraine attack is proposed to result in sensitization of trigeminal nociceptors, potentially explaining hyperalgesia and allodynia^3^. Pain perceived during a migraine attack is mainly located within the innervation area of the trigeminal nervous system^4^.

Migraine is characterized by attacks lasting 4 to 72 h, moderate to severe pain intensity, unilateral localization, and pulsatile pain. It is further associated with photophobia and phonophobia, nausea, and often worsens with physical activity^5^. Another highly prevalent yet often neglected and much-discussed manifestation of migraine is neck pain^6,7^. Up to 77% of migraineurs have neck pain, making it as frequent as all associated symptoms mentioned above. Compared to headache-free people, patients with migraine are 12 times more likely to have neck pain^8^. Nevertheless, neck pain is not part of the migraine diagnostic criteria^5^.

Neck pain in patients with migraine is related to a more severe clinical presentation of migraine attacks and worse migraine-related disability^9^. It is seen as a potential trigger for migraine attacks, correlates with a higher attack frequency, and is associated with a higher risk of chronicity^10–12^. Hence, the clinical relevance of neck pain and the need for a standard evaluation of the cervical spine of patients is evident. In addition, patients with migraine have a significantly higher presence of cervical musculoskeletal dysfunctions^7^. Further, there is an ongoing scientific debate as to whether neck pain should be considered a migraine symptom or whether, if it is due to a neck dysfunction, and if it may be a cause of or contributing factor to migraine.

A collection of standard headache assessment tests (HATs) for evaluating musculoskeletal neck dysfunctions in patients with migraine has been presented by Luedtke et al.^13^. Another commonly used and highly validated tool for assessing neck-related disability is the Neck Disability Index (NDI)^14^. Of all tests of the HATs, particularly manual joint palpation and trigger point palpation, had the highest prevalence of findings in migraine patients^7^. Luedtke and May continued investigating these findings by palpation of the upper cervical segments. With the upper cervical palpation test (UPT), patients can be stratified into three groups of neck pain perception, which may have high clinical relevance for future treatment of migraine: no pain, local pain, and referred pain during sustained palpation^6^.

Manual examination via the UPT requires pressure through suboccipital structures (joints, ligaments, suboccipital muscles). Hence, pain provocation during palpation could indicate either muscle or joint changes^6^. The trigeminocervical convergence theory represents a possible interaction between the musculoskeletal upper cervical and trigeminal nerve systems, as these are closely connected anatomically^4,15^. Considering this, the UPT may provide a mechanical explanation for local and referred pain sensitivity during the examination, as palpation is performed directly at the atlantooccipital and atlantoaxial joints^6^. These findings of different pain sensations in the upper cervical spine may indicate the presence of cervical musculoskeletal changes in migraine patients. However, it is not clear yet whether the neck pain in migraine patients is caused by a generally more sensitized system due to peripheral or central sensitization or by muscle or joint-related neck-related disability.

Since neck pain and disability in migraine patients have been physically assessed with the UPT^6^, there needs to be further clarification of what the test truly evaluates. Therefore, the aim of the study was to assess the differences of the UPT subgroups regarding sensitization and neck pain/related disability. Besides, to determine whether upper cervical spine sensitivity tested by the UPT is associated with neck-related disability or increased pain sensitization in migraine patients. The first hypothesis is that the UPT may be explained by neck pain and/or neck-related disability. The second hypothesis is that the UPT may be explained by central sensitization.

## Methods

The study protocol was preregistered on the Open Science Framework Platform (10.17605/OSF.IO/CTKHM) and performed in accordance with relevant guidelines/regulations. The study was approved by the ethics committee of the University of Luebeck. All participants provided written informed consent prior to the data collection.

### Participants

This study is part of the “MigrainePREDICT” study, in which at least 38 patients with migraine were required. Participants were recruited by convenience sampling from the campus of the University of Luebeck and social networks and the inclusion criteria were age between 18 and 73 years old, episodic migraine with or without aura with at least two headache days per month, fulfilling the diagnostic criteria of the third International Classification of Headache Disorders (ICHD-3)^5^. Participants were excluded from the study if they had a headache on the day of the examination, took pain medication on the day of the examination, or had any additional chronic systemic, neurological, or psychiatric illness. Further exclusion criteria were diagnosed acute neck pathologies or neck injuries such as rear-end collision or whiplash injuries in the last ten years.

### Measurements

All participants filled out a questionnaire on demographic data and migraine- and neck-specific characteristics. Questions included age, migraine pain intensity, migraine attacks per month, and migraine diagnosis confirming associated symptoms such as an increase of the symptoms with activity, light, or noise. The presence of neck pain, its intensity and frequency were also recorded.

### Upper cervical palpation test

**Fig. 1:**
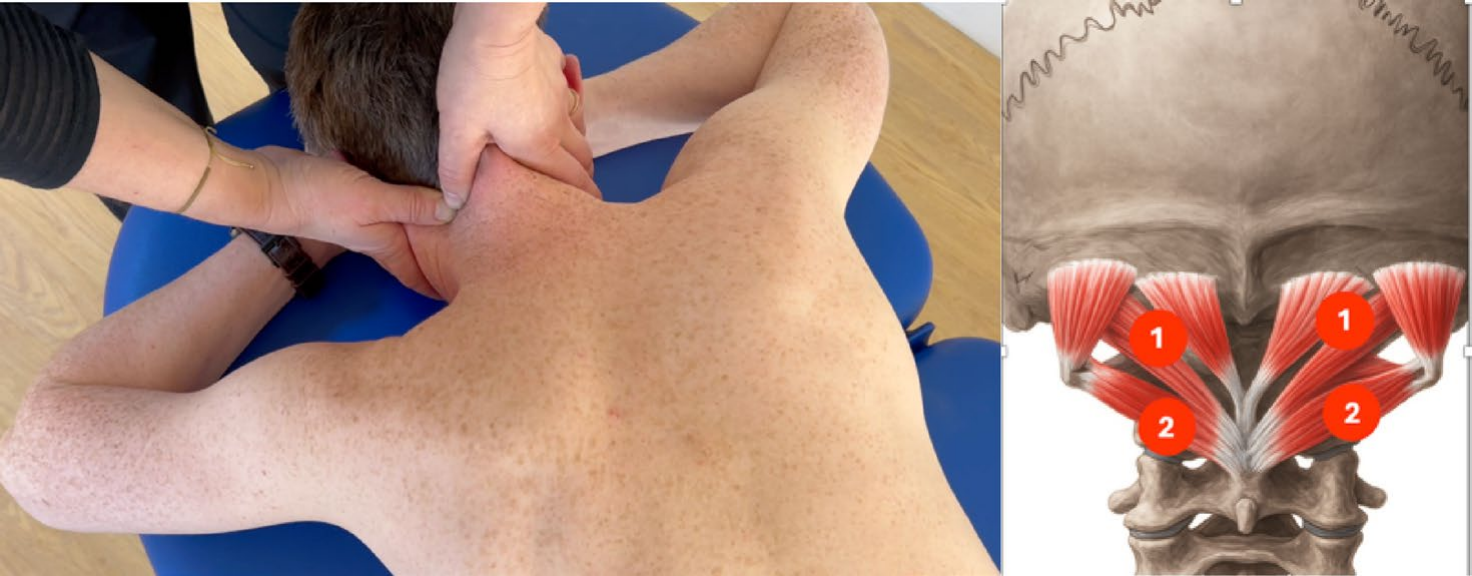
Upper cervical palpation test.

Upper cervical spine sensitivity was tested using the UPT, described by Luedtke and May^6^ (Fig. 1). Oscillating and sustained palpation for about 30 s was performed on both sides of the transverse process of C1 (testing the left and right atlantooccipital joint number 1 on Fig. 1), and on both sides of the transverse process of C2, testing the left and right atlantoaxial joint (number 2 on Fig. 1). Patients were positioned in prone with their hands supporting their forehead. Pain intensity was verbally reported on a numerical pain scale from 0 to 10. Pain location was assessed as “no pain” if they did not feel any pain perception, “local pain” for pain in the neck area, and “referred pain” for pain referring to the head or back. The examiner further documented whether hypomobility was present in the tested joints (binary outcome).

### Neck disability and intensity

For measuring neck-related disability, all participants completed the German version of the Neck Disability Index (NDI), which is a valid and reliable 10-item questionnaire on self-reported disability due to neck pain, with each question scored from 0 to 5 and a total score ranging from 0 to 50^14,16,17^. The NDI was implemented and validated for neck pain associated with various cervical musculoskeletal conditions^14,16,18–20^ Neck pain intensity was additionally self-evaluated in the demographic and symptom questionnaire using a numerical pain rating (NPRS) scale from 0 to 10^21^.

### Central sensitization

For measuring central sensitization, all participants completed the Central Sensitization Inventory (CSI)^22^ part A. Part A is a questionnaire assessing 25 symptoms, on a scale from 1 to 4, associated with central sensitization syndrome with a total score ranging from 0 to 100. Part B was not used for this study as it focuses on additional diagnoses (e.g. Fibromyalgia, Chronic Fatigue Syndrome) related to central sensitization, which were exclusion criteria for this study^23^. In addition, the Allodynia Symptom Checklist (ASC-12)^24^ was evaluated, a 12-item questionnaire with total scores ranging from 0 to 24 that asks about the presence and frequency of allodynia symptoms during a migraine attack and thus measures cutaneous allodynia.

### Mechanical pain threshold and pressure pain threshold

The mechanical pain threshold (MPT) and pressure pain threshold (PPT) of the standardised and validated quantitative sensory testing (QST) protocol^25^ were assessed bilaterally to further quantify sensitivity. MPT and PPT were used at four sites: the neck (spinous process of C2), a receptive field of the first branch of the trigeminal nerve (V1) (above left and right eyebrow), and at a remote site (outside the neck or the receptive field of the trigeminal nerves (arm).

At all sites, MPT was measured with standardised PinPrick stimulators weighted from 8 to 512 mN (MRI Compatible Pinprick Stimulator Set; MRC Systems GmbH, Heidelberg, Germany). At each site, three series of threshold determination were performed, using ascending and descending stimuli until the participant reported a “sharp” or “blunt” sensation. There was no fixed time interval between the repetitions, but a brief pause was allowed to reposition the equipment and ensure consistency. MPT values were transformed logarithmically and calculated as the geometric mean. A higher mean value is associated with lower mechanical sensitivity.

PPTs were obtained with a standardised mechanical pressure algometer with a 1 cm^2^ contact surface and a 1–10 kg/cm^2^ scale (Force Dial FDK/FDN 20 algometer, Wagner Instruments, Greenwich, USA). At each site, threshold determination was performed three times by increasing the pressure by 0.5 k/s until participants reported the first sensation of pain. There was no fixed time interval between the repetitions, but a brief pause was allowed to reposition the equipment and ensure consistency and to avoid temporal summation of pain. PPT values were transformed logarithmically and calculated as the geometric mean. A higher mean value is associated with lower pressure sensitivity.

### Procedures

All eligible and included participants (n = 42) first completed a series of questionnaires. Participants who were included were subsequently assigned to UPT and quantitative sensory testing for the MPT and PPT in a randomized order. All examiners were blinded toward migraine diagnosis, as the examination was part of a larger study, which also recruited healthy control participants. After all tests, the participants were stratified into three groups according to the results of the UPT (no pain, local pain, referred pain)^6^.

The examiner to the UPT test was a licensed physiotherapist with over seven years of clinical experience, and in the past six years, has been treating patients with headaches. Additionally, prior to data collection, all examiners underwent training and practice toward all assessments to ensure consistency. All participants were assessed using the same procedures to minimize potential bias.

### Statistical analysis

Statistical analyses were performed with JAMOVI with a significance level of 5%. Demographic data and questionnaires for headache and neck pain characteristics were presented for each pain group as mean values, standard deviations, and percentages. To assess the normality of the data, Shapiro–Wilk test was performed. MPT and PPT results were transformed logarithmically prior to statistical analysis and are presented as mean values and standard deviations. Differences between UPT subgroups, regarding all test results, were assessed by Kruskal–Wallis-Test and chi-square test to account for the small sample sizes in each subgroup. Multinominal logistic regression model was performed with UPT groups (no pain, local pain and referred pain) and multilinear regression models with the NDI and CSI total scores as dependent variable, considering the variables that had significant associations with the dependent variable (*p* < 0.200) to explain which factors contribute to each score. To further explore correlations between the variables, Pearson’s correlation test was used for interval-scaled data and Spearman’s rho for ordinal-scaled data.

## Results

**Fig. 2:**
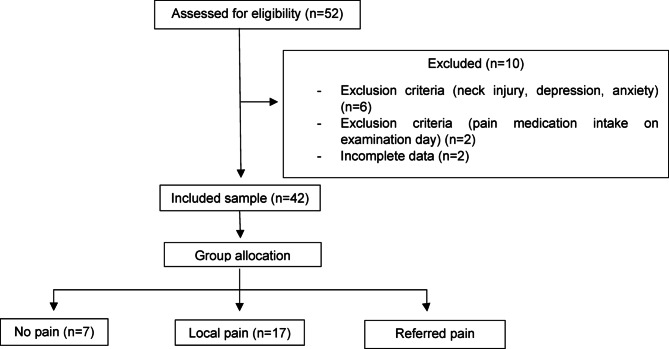
Flow diagram of study.

A total of 52 migraine patients were recruited for the study. Ten individuals were excluded as they did not fulfil the inclusion criteria. The 42 eligible participants were included in the analysis and were stratified into three UPT subgroups (Fig. 2).

Demographic data, headache, and neck pain characteristics of the participants for each subgroup of the UPT, are represented in Table 1. No statistically significant differences were found among the subgroups considering all measurements of neck-related disability and central sensitization (Tables 1 and 2).

**Table 1:** Participant characteristics and group differences.

	No pain (n = 7)		Local pain (n = 17)		Referred pain (n = 18)		X^2^ (df)	*P* value
	Mean or percentage	SD	Mean or percentage	SD	Mean or percentage	SD		
Demographic data and questionnaires								
Age (years)	33.2	13.3	33.9	12.8	32.6	13.1	0.24 (2)	0.885
Sex (female)	85.7%		88.2%		72.2%		1.58 (2)	0.454
NDI	7.3	3.2	6.4	3.9	7.9	3.3	1.57 (2)	0.456
ASC12	4.6	4.4	4.9	3.7	3.9	2.4	0.55 (2)	0.759
CSI	29.0	7.2	34.7	11.4	36.5	8.5	3.58 (2)	0.167
UPT total pain intensity	–	–	2.2	2.1	2.5	2.0		
Headache characteristics								
Headache pain intensity (NPRS)	8.1	1.3	7.1	2.6	7.4	3.2	1.41 (2)	0.493
Migraine attacks per month	3.6	3.0	2.6	1.5	2.3	1.5	2.11 (2)	0.349
Associated symptoms								
Physical activity	71.4%		88.2%		83.3%			
Light or noise	100%		88.2%		100%			
Nausea/vomiting	71.4%		70.6%		61.1%			
Neck pain characteristics								
Presence of neck pain	85.7%		82.4%		77.8%		0.23 (2)	0.888
Pain intensity (NPRS)	5.1	2.8	4.8	2.7	4.5	2.2	0.076(2)	0.963
Pain frequency per month								
Never	14.3%		17.7%		22.2%			
Sometimes	14.3%		17.7%		11.1%			
2–3 times a month	28.6%		41.2%		22.2%			
4 times a month	42.9%		5.9%		11.1%			
> 4 times a month	0.0%		11.8%		22.2%			
Daily	0.0%		5.9%		11.1%			

Mean values and standard deviations (SD) of demographic data, headache, and neck pain characteristics, questionnaires for the three groups “no pain (n = 7)”, “local pain (n = 17)”, and “referred pain (n = 18)”. *P*-values of group differences by Kruskal-Walli testing and chi-square testing. NDI: neck disability index; CSI: central sensitization inventory; ASC-12: allodynia symptom checklist 12.

**Table 2:** iParticipant characteristics and group differences regarding MPT/PPT.

	No pain (n = 7)		Local pain (n = 17)		Referred pain (n = 18)		Group differences	
	Mean	SD	Mean	SD	Mean	SD	Χ^2^ (df)	*p*-value
PPT neck	0.4	0.3	0.4	0.3	0.4	0.1	0.62 (2)	0.734
PPT left eyebrow	0.3	0.1	0.4	0.1	0.4	0.1	0.30 (2)	0.860
PPT right eyebrow	0.4	0.2	0.4	0.1	0.4	0.1	0.03 (2)	0.984
PPT arm	0.5	0.3	0.5	0.2	0.5	0.1	0.05 (2)	0.974
MPT neck	1.6	0.5	1.5	0.5	1.4	0.5	0.29 (2)	0.861
MPT left eyebrow	1.6	0.6	1.3	0.5	1.4	0.4	1.86 (2)	0.396
MPT right eyebrow	1.7	0.5	1.5	0.5	1.4	0.5	1.85 (2)	0.397
MPT arm	1.4	0.4	1.2	0.3	1.4	0.5	1.07 (2)	0.585

Log and standard deviations (SD) for the results of pressure pain threshold (PPT) and mechanical pain threshold (MPT) for the three groups “no pain (n = 7)”, “local pain (n = 17)”, and “referred pain (n = 18)”. *P*-values of group differences by Kruskal–Wallis testing.

PPT, pressure pain threshold; MPT, mechanical pain threshold.

There were no significant correlations among the CSI and all MPT and PPT results (*p* > 0.05). Therefore, these variables were not used for further statistical analysis (Table 3).

**Table 3:** Pearson’s correlation CSI and MPT/PPT.

	MPT					PPT				*P* value
	Arm	Neck	Right eyebrow		Left eyebrow	Arm	Neck	Right eyebrow	Left eyebrow	
CSI	− 0.189	0.024	− 0.035	− 0.123		0.077	− 0.107	0.027	− 0.001	> 0.05

Pearson’s correlation considering the CSI and all MPT/PPT variables.

CSI, central sensitization inventory; PPT, pressure pain threshold; MPT, mechanical pain threshold.

For the logistic regression considering the UPT subgroups as the dependent variable, the reference category was the no pain group (0), number 1.00 was the local pain group, and number 2.00 was the referred pain group. The model was not significant (X^2^ = 10.540(8); *p* = 0.229), therefore even though the CSI was a significant predictor, this result cannot be considered (Table 4).

**Table 4:** Multinomial Logistic Regression (UPT as dependent variable).

UPT	Model	R^2^	R^2^ adjusted	Unstandardized coefficients		Sig		95%CI
				β	St. Error		Exp(B)	
1.00	Constant	0.229	0.379	− 1.31	1.77	0.459		
	CSI			0.17	0.86	**0.049**	1.19	1.001 to 1.404
	ASC-12			− 0.12	0.17	0.477	0.89	0.638 to 1.234
	NDI			− 0.35	0.24	0.149	0.71	0.439 to 1.133
	Neck pain intensity			− 0.28	0.04	0.488	0.97	0.898 to 1.053
2.00	Constant	0.229	0.379	− 1.95	1.73	0.259		
	CSI			0.18	0.09	**0.039**	1.20	1.010 to 1.422
	ASC-12			− 0.27	0.18	0.132	0.76	0.537 to 1.085
	NDI			− 0.21	0.24	0.391	0.81	0.504 to 1.307
	Neck pain intensity			− 0.03	0.04	0.374	0.97	0.899 to 1.404

Multinomial Logistic Regression with the UPT as a dependent variable and the variables related to central sensitization and neck-related disability.

UPT, upper cervical palpation test; Reference category, no pain group; CSI, central sensitization inventory; ASC-12, allodynia symptom checklist 12; NDI, neck disability index; St. Error, standard error; Sig, level of significance.

Significant values are in bold.

Subsequently, it was evaluated which of the variables influenced neck disability and central sensitization, respectively. In Table 5, considering the NDI score as the dependent variable, the results showed that model 3 was statistically significant [F = 16.870; *p* = 0.000; R^2^ adjusted = 0.436] and that NDI, was explained to 43.6% by the CSI questionnaire (*p* = 0.000) and neck pain intensity (*p* = 0.032).

**Table 5:** Linear regression with the NDI as a dependent variable.

Model		R^2^	R^2^ adjusted	Unstandardized coefficients	Standardized coefficients		Sig	Collinearity statistics	
				β	St. Error	Beta		Tolerance	VIF
1	Constant	0.47	0.410	− 0.69	1.61		0.667		
	ASC-12			− 0.71	0.15	− 0.07	0.631	0.8	1.3
	CSI			0.23	0.05	0.63	**0.000**	0.7	1.4
	Neck pain intensity			0.07	0.04	0.25	0.056	0.9	1.1
	UPT			− 0.13	0.62	− 0.03	0.835	0.9	1.2
3	Constant	0.46	0.436	− 0.78	1.53		0.613		
	CSI			0.22	0.04	0.59	**0.000**	0.9	1.0
	Neck pain intensity			0.07	0.03	0.26	**0.032**	0.9	1.0

Linear regression with the Neck Disability Index (NDI) as a dependent variable and the variables related to central sensitization and neck-related disability.

UPT, upper cervical palpation test; CSI, central sensitization inventory; ASC-12, allodynia symptom checklist 12; St. Error, Standard Error; Sig, level of significance; VIF, Variance Inflation Factor.

Significant values are in bold.

Considering the CSI score as the dependent variable, the results demonstrated that model 2 was statistically significant [F = 14.328; *p* = 0.000; R^2^ adjusted = 0.494]. CSI was explained in 49.4% by the ASC-12 (*p* = 0.006) and the NDI (*p* = 0.000). The UPT did not significantly explain the CSI (*p* = 0.056), as shown in Table 6.

**Table 6:** Linear regression with the CSI as a dependent variable.

Model		R^2^	R^2^ adjusted	Unstandardized coefficients		Standardized coefficients	Sig	Collinearity statistics	
				β	St. Error	Beta		Tolerance	VIF
1	Constant	0.53	0.480	15.5	3.29		0.000		
	ASC-12			0.96	0.35	0.37	**0.009**	0.9	1.1
	NDI			1.53	0.34	0.56	**0.000**	0.8	1.2
	Neck pain intensity			− 0.00	0.09	− 0.00	0.988	0.8	1.2
	UPT			2.95	1.54	0.22	0.063	0.9	1.1
2	Constant	0.53	0.494	15.5	3.23		0.000		
	ASC-12			0.96	0.33	0.33	**0.006**	0.9	1.0
	NDI			1.53	0.31	0.55	**0.000**	0.9	1.0
	UPT			2.95	1.49	0.22	0.056	0.9	1.0

Linear regression with the Central Sensitization Inventory as a dependent variable and the variables related to central sensitization and neck-related disability.

UPT, upper cervical palpation test; NDI, neck disability index; ASC-12, allodynia symptom checklist 12; St. Error, Standard Error; Sig, level of significance; VIF, Variance Inflation Factor.

Significant values are in bold.

## Discussion

The purpose of this study was to disentangle neck-related disability or pain sensitivity as explanations for UPT results in patients with migraine.

In the sample, 83% percent of migraine patients perceived local or referred neck pain during the UPT. Seventeen percent had no pain during the examination, and 43% perceived referred pain to the head. These results corroborate the UPT findings of Luedtke et al.^6^, who reported that 80% of migraineurs perceived pain on upper cervical spine palpation, with half of these also reporting referred pain. It, however, shows a different proportion of Watson and Drummond^26^ who observed higher rates of head pain referral in both migraine and tension-type headache populations or Liang et al.^27^ that showed much lower number of cervical musculoskeletal findings in patients with migraine, compared with tension type headache. Differences between these reports can only be hypothesized and are most likely based on either the population characteristics, the purpose of the studies or individual differences across examiners.

The subgroup stratification according to the UPT may indicate clinically relevant migraine subtypes: migraine patients without UPT neck findings may experience neck pain as a migraine symptom without any ongoing nociception from the upper cervical spine. In contrast, migraine patients with UPT findings might have upper cervical spine dysfunctions, which could be contributing to migraine-associated disability^6^.

Considering that the UPT requires pressure on suboccipital structures and assesses the patient’s pain response, results may either indicate local muscle or joint changes^6^ or evaluate general pain sensitivity, potentially influenced by central sensitization processes.

In this current sample, UPT subgroups did not differ significantly regarding neck disability or neck pain intensity, CSI, ASC-12, and MPT and PPT measures. This is line with the results of the observational study by Schwarz et al. (2021), in which no statistically significant subgroup differences were found regarding neck-related disability or allodynia (NDI and ASC-12). This raises the question of how the different pain perceptions, leading to the subgroup allocation, can be explained**.**

The most obvious hypothesis is that the UPT assesses musculoskeletal neck dysfunction and that subgroup allocations could, hence, also be explained by NDI, and neck pain intensity. However, this hypothesis could not be confirmed. In fact, the “no pain” group of the UPT reported higher levels of average neck pain intensity than the “local and referred pain” groups. There are several ways to explain these results: The overall prevalence of neck pain in this current sample was very high (82%) and highest in the “no pain” UPT group.

The UPT assesses the upper cervical spine (C1 and C2), as these cervical segments are assumed to influence the trigeminal nervous system through proximity of spinal and trigeminal afferents within the brainstem^4^. Schwarz et al. further elaborated that only the upper two cervical segments may contribute to pain provocation in patients with migraine^28^.

The literature on neck pain, however, reports that the majority of neck pain and disability are caused by mid and lower cervical segments^29^. It, therefore, seems likely that the neck pain and disability reported by this current sample are independent phenomena alongside migraine, affecting lower parts of the cervical spine, which were not assessed during the UPT. This explanation aligns with Liang et al., who postulated that the NDI–when used in migraine populations–is more influenced by allodynia or central sensitization than by musculoskeletal dysfunction since, in their study, a correlation between NDI and cervical dysfunction could not be found. This suggestion is potentially confirmed by the results of this study, in which the NDI was explained by 43.6% for the CSI questionnaire and neck pain intensity.

The second hypothesis, that UPT subgroups are determined by the level of central sensitization, was also not confirmed. Even for the highest CSI values in the “referred pain” group and lowest in the “no pain” group, the ANOVA analyses did not provide statistically significant differences between subgroups regarding indicators for central sensitization (CSI, ASC-12, and mechanical and pressure pain thresholds).

Less surprisingly, CSI and ASC-12 showed a positive and statistically significant correlation- this indicates that the perceived unpleasantness of sensory stimuli in the face and neck during migraine attacks (as measured by the ASC-12) is related to an overall level of increased sensitivity and perceived unwellness (measured by the CSI).

The lack of correlation between the QST variables and CSI is more challenging to interpret, given that previous studies by Mibu et al. and Steinmetz et al. reported a high correlation between PPTs and CSI^30–32^. However, these studies were not performed in migraine populations but in chronic low-back pain, knee osteoarthritis, and musculoskeletal disorders^30–32^. Regarding migraine, our findings diverge from previous studies that have demonstrated significant associations between central sensitization and sensory processing in migraine^33,34^. A potential explanation for this discrepancy lies in our smaller sample size and in methodological differences, such as the stratification of the participants across different phases of the migraine cycle^33^. Furthermore, approximately one-third of the items on the CSI are typically reported associated symptoms of migraine attacks, such as headache, temporomandibular symptoms, nausea, sleep, cognition, and sensitivity to light^35^. Hence, the CSI results recorded in this current sample might reflect more typical migraine-associated symptoms than central sensitization.

One important limitation of the current study is that it was not systematically controlled for the migraine cycle phase at the time of the assessment, since the participants were assessed at a single time point without migraine phase classification, however, the purpose was not to differentiate between migraine phases but to assess the influences on UPT test results. Since pain sensitivity is expected to change with the migraine phases the association between sensitivity and UPT results should remain stable.

This study only investigated C1 and C2 and did not manually assess mid or lower cervical segments or use other tests. However, previous research showed that the NDI and manual palpation results are correlated^24^. It can, therefore, be postulated that the neck-related disability reported by this study population were explained by musculoskeletal dysfunction in the lower parts of the cervical spine.

No imaging or additional tests were performed to assess the upper cervical spine, which could confirm the UPT results. Hence, the results must be interpreted with caution and can only exclude the influence of central sensitization and perceived neck-related disability as driving factors for subgroup classification.

The sample sizes of the UPT subgroups were relatively small^6,34^. However, similar group sizes are used in other studies on the UPT subgrouping. Furthermore, Kruskal–Wallis testing is robust even in small sample sizes^36^.

## Conclusion

The current results confirm the high prevalence of neck pain in migraineurs and of pain during UPT reported in previous studies within this population. Pain on UPT is not determined by central sensitization, as measured by CSI or by overall neck-related disability, as measured by the NDI. Hence, the NDI seems to be of limited use in detecting upper cervical musculoskeletal dysfunction in patients with migraine. Similarly, the CSI seems to provide limited information on central sensitization in migraine patients since one-third of the items describe migraine-related symptoms. The UPT, therefore, is believed to measure an independent construct, most likely upper cervical musculoskeletal dysfunction that should be confirmed in future studies.

## Data Availability

The data that support the findings of this study are available on request from the corresponding author, KL.
